# Study on the Influence of Sn Concentration on Non-Substitutional Defect Concentration and Sn Surface Segregation in GeSn Alloys

**DOI:** 10.3390/molecules30091875

**Published:** 2025-04-23

**Authors:** Zihang Zhou, Jiayi Li, Mengjiang Jia, Hai Wang, Wenqi Huang, Jun Zheng

**Affiliations:** 1 School of Applied Science, Beijing Information Science and Technology University, Beijing 102206, China; 2023021107@bistu.edu.cn (M.J.); 2024021175@bistu.edu.cn (H.W.); 2 Key Laboratory of Optoelectronic Materials and Devices, Institute of Semiconductors, Chinese Academy of Sciences, Beijing 100083, China; lijiayi@semi.ac.cn (J.L.); zhengjun@semi.ac.cn (J.Z.); 3 College of Materials Science and Opto-Electronic Technology, University of Chinese Academy of Sciences, Beijing 100049, China

**Keywords:** GeSn alloys, substitutional Sn, non-substitutional Sn defects, surface segregation, formation energy

## Abstract

GeSn alloys are among the most promising materials for the fabrication of high-efficiency silicon-based light sources. However, due to the tendency of Sn to segregate to the surface during growth, it is challenging to achieve a high Sn concentration while maintaining high-quality GeSn alloys. Both theoretical and experimental studies have confirmed that non-substitutional Sn defects (VSnV) are the primary driving factors in Sn surface segregation. However, there is a discrepancy between existing theoretical and experimental findings regarding the variation in VSnV concentration with total Sn concentration. To clarify this issue, we first prepared GeSn materials with varying Sn concentrations using molecular beam epitaxy (MBE) and subjected them to annealing at different temperatures. Subsequently, we characterized the VSnV concentration and Sn surface segregation. The results indicate that a higher total Sn concentration and temperature lead to an increased VSnV concentration, and the proportion of VSnV relative to the total Sn concentration also increases, which is consistent with existing theoretical research. To explain these phenomena, we employed first-principles calculations based on density functional theory (DFT) to investigate the effect of varying the total Sn concentration on the formation of substitutional Sn (Sn_s_) and VSnV in GeSn alloys, while simultaneously studying the migration kinetics of Sn atoms. The results demonstrate that as the total Sn concentration increases, the formation of Sn_s_ becomes more difficult, while the formation of VSnV becomes easier, and Sn atoms exhibit enhanced migration tendencies. The analysis of binding energies and charge density distribution maps reveals that this is due to the weakening of Ge-Sn bond strength with increasing Sn concentration, whereas the binding strength of VSnV exhibits the opposite trend. These findings demonstrate excellent agreement with experimental observations. This study provides both theoretical and experimental references for GeSn material growth and VSnV defect control through a combined theoretical–experimental approach, offering significant guidance for enhancing device performance.

## 1. Introduction

In recent years, significant progress has been made in silicon-based optical interconnect systems, with the successful development of photodetectors, optical waveguides, optical amplifiers, optical modulators, and optical switches [1,2,3]. However, the development of an efficient silicon-based light source remains an unresolved challenge [4]. This is primarily due to the fact that among the group IV elements compatible with silicon, silicon (Si) and germanium (Ge) are indirect-bandgap materials, while tin (Sn) and lead (Pb) are zero-bandgap materials [5]. The fabrication of efficient silicon-based light sources requires materials with a direct bandgap. GeSn alloys can achieve a direct bandgap by adjusting the Sn concentration, offering new hope for addressing this challenge. Theoretical studies predict that relaxed GeSn undergoes a direct bandgap transition at Sn concentrations ranging from 6.5% to 11.0% [6,7,8,9], while experimental studies demonstrate that strained GeSn alloys achieve this transition at a Sn concentration of 12% [6]. However, the Sn concentration should not be excessively high, as the bandgap of GeSn approaches zero when the Sn concentration reaches approximately 25% [7]. To date, researchers have developed both optically pumped [6,10] and electrically pumped [11] GeSn lasers, marking significant advancements toward achieving silicon-based optical interconnects [12,13]. Nevertheless, electrically pumped GeSn lasers are still far from achieving practical room-temperature lasing due to their low lasing temperatures and high threshold currents [14,15]. The growth of high-quality GeSn alloys with high Sn concentrations is crucial for achieving room-temperature electrically pumped lasing [16]. This is because a higher Sn concentration results in a larger energy difference between the Γ-valley (direct band valley) and L-valley (indirect band valley) in the conduction band of GeSn. This facilitates the accumulation of electrons in the Γ-valley, significantly enhancing the optical gain from electron transitions [17], thereby increasing the lasing temperature and reducing the threshold current [6,11,15].

However, the surface segregation of Sn during the growth process poses one of the most significant challenges in achieving high-quality and high-Sn-concentration GeSn alloys, which is also a primary factor affecting device performance [12,18,19]. The surface segregation of Sn is closely related to the clustering states of Sn atoms. In GeSn crystals, the primary Sn clusters consist of Sn-Sn clusters and Sn–vacancy (Sn-V) clusters. Ventura et al. [20] demonstrated that the formation of Sn-Sn clusters is highly unfavorable, a conclusion further supported by computational studies conducted by Karthikeyan et al. [21]. Since Sn migration in Ge follows a vacancy-mediated mechanism [22,23], Sn atoms preferentially combine with V to form Sn-V clusters. Experimental measurements by Decoster, Assali, Timofeev, and Zaima et al. [24,25,26,27] confirm that non-substitutional Sn defects (VSnV) are the dominant cluster defects in as-grown GeSn layers. Theoretical studies by Ventura [20] and experimental investigations by Decoster [26] have revealed that VSnV in GeSn alloys are the primary driving factors in Sn surface segregation. This phenomenon occurs because, after Sn incorporates into the Ge lattice, the majority of Sn atoms substitute Ge atoms to form substitutional Sn (Sn_s_), while a portion of Sn atoms combine with two vacancy defects to form VSnV, which exhibits a quasi-cubic octahedral structure similar to white tin. VSnV and Sn_s_ create opposing force fields within the GeSn lattice, thereby promoting the segregation of Sn to the surface. Consequently, elucidating the variation in VSnV concentrations is crucial in understanding the formation mechanisms of VSnV and, ultimately, the extent of Sn segregation.

However, there is a discrepancy between existing theoretical and experimental findings regarding the variation in VSnV concentration with total Sn concentration. Theoretical studies by Ventura et al. [20] suggest that the concentration of VSnV increases with the total Sn concentration, and the ratio of VSnV to the total Sn concentration also rises. In contrast, Decoster et al. [26] introduced Sn into Ge crystals using ion implantation and measured the concentration of Sn_s_ and VSnV using emission channeling techniques. Their results indicate that the proportion of VSnV concentration remains relatively constant across different total Sn concentrations and annealing temperatures, which contradicts the theoretical predictions of Ventura et al. This inconsistency arises because the GeSn alloys generated by Decoster et al. through ion implantation exhibit poor homogeneity and significant lattice damage. This method is only suitable for low-concentration localized doping, rendering their experimental results inaccurate. Asali et al. [25] employed the more advanced chemical vapor deposition (CVD) method to grow GeSn alloys and confirmed VSnV as the predominant defect in GeSn. However, their study did not investigate the variation in VSnV concentration with total Sn concentration. Furthermore, their characterization of VSnV defects through the combined use of depth-profiled positron annihilation lifetime spectroscopy (PAS) and Doppler broadening measurements represents a relatively expensive analytical approach. Furthermore, the underlying physical reasons for these theoretical and experimental results have not yet been explored in the literature.

Building upon the aforementioned analysis, this study adopts a combined experimental and theoretical approach to addressing these issues. Initially, the advanced molecular beam epitaxy (MBE) technique is employed to synthesize GeSn alloys with a controlled Sn concentration. Subsequently, we implement more economical and effective characterization methods to comprehensively evaluate, from both quantitative and qualitative perspectives, the variation patterns of Sn_s_ and VSnV concentrations as functions of the total Sn concentration and temperature. Finally, first-principles calculations based on density functional theory (DFT) are performed to determine the formation energies, binding energies, and charge density distributions of Sn_s_ and VSnV. These computational analyses aim to provide a deeper understanding of the underlying physical mechanisms driving the observed experimental phenomena, thereby offering insights into defect formation and stability in GeSn alloys.

## 2. Results and Discussion

### 2.1. Material Characterization and Analysis

**Figure 1:**
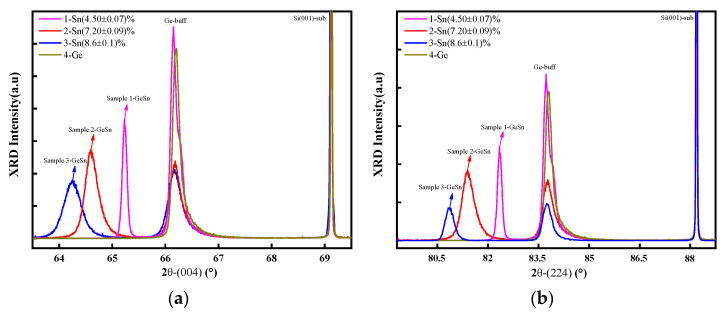
(**a**) The XRD spectrum of the GeSn/Si(001) samples in (004) direction. (**b**) The XRD spectrum of the GeSn/Si(001) samples in (224) direction.

Firstly, GeSn materials with varying Sn concentration were fabricated on Si(100) substrates, designated as samples 1, 2, and 3. X-ray diffraction (XRD) characterization was performed along the (004) and (224) crystallographic orientations, as shown in Figure 1. It can be observed that the GeSn diffraction peaks for samples 1 to 3 are relatively symmetric, indicating a high degree of material homogeneity. Accordingly, the diffraction peaks of GeSn shift to the left (toward lower angles), demonstrating the successful incorporation of Sn into the lattice. According to Bragg’s law, this shift is due to the increase in the lattice constant of GeSn alloys with higher Sn concentrations. Since the coordination environments of Sn_s_ and VSnV differ, the concentration of Sn_s_ significantly influences the lattice constant of GeSn, whereas the effect of VSnV concentration is minimal [20]. Therefore, the concentration of Sn_s_ can be determined from the variation in lattice constants. In our measurements, multiple experimental repetitions were performed, and the average values were adopted as final results to minimize experimental errors. All subsequent measurements followed this standardized methodology to ensure data consistency and reliability. Based on the peak shifts in the Ge-Sn diffraction, the Sn_s_ concentration in samples 1, 2, and 3 was calculated to be (4.50 ± 0.07)%, (7.20 ± 0.09)%, and (8.6 ± 0.1)%, respectively. At a lower concentration, the GeSn diffraction peaks exhibit a narrow full width at half maximum, indicating superior crystal quality. As the Sn_s_ concentration increases, the GeSn diffraction peaks gradually broaden, suggesting a degradation in the crystallographic quality of the material.

**Figure 2:**
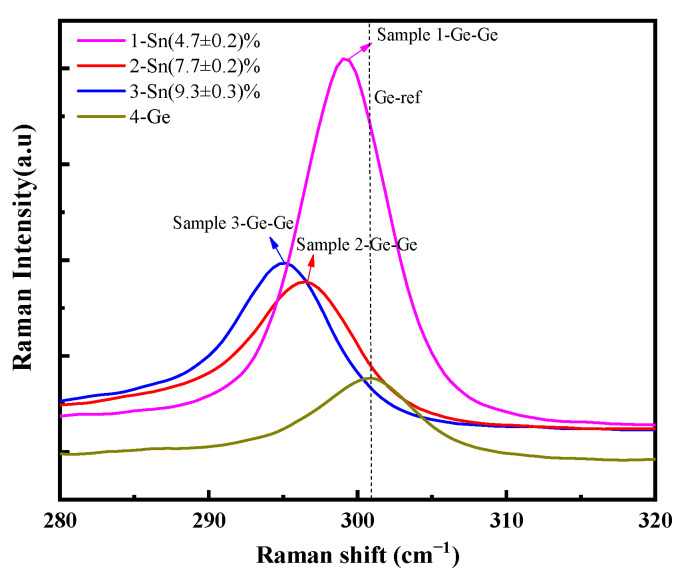
The Raman spectrum of the GeSn/Si(001) samples.

The Raman spectra of the samples were also measured, as shown in Figure 2. It can be observed that the Ge-Ge peaks of samples 1, 2, and 3 exhibit a shift to the left, which is attributed to the increase in Sn_s_ concentration. Based on the shift in the Ge-Ge resonant peaks, the Sn_s_ concentration of samples 1, 2, and 3 was determined to be (4.7 ± 0.2)%, (7.7 ± 0.2)%, and (9.3 ± 0.3)%, respectively. These results are in good agreement with those obtained from XRD calculations. Furthermore, our analysis revealed that Raman spectroscopy exhibited larger measurement uncertainties compared to XRD; therefore, we employed Raman measurements as a semi-quantitative analytical approach to complement our primary characterization methods.

**Figure 3:**
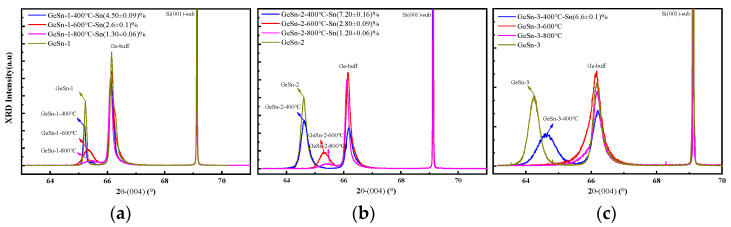
(**a**) The XRD spectrum of the GeSn/Si(001) sample 1 at different annealing temperatures in (004) direction; (**b**) the XRD spectrum of the GeSn/Si(001) sample 2 at different annealing temperatures in (004) direction; (**c**) the XRD spectrum of the GeSn/Si(001) sample 3 at different annealing temperatures in (004) direction.

Subsequently, annealing experiments were conducted on the samples. Figure 3 displays the XRD spectra of samples 1, 2, and 3 annealed at temperatures of 400 °C, 600 °C, and 800 °C. It can be observed that at an annealing temperature of 400 °C, samples 1 and 2 exhibit good stability, while the peak position of sample 3 shifts to the right, indicating that GeSn alloy samples with a higher Sn concentration possess inferior thermal stability compared to those with a lower Sn concentration. However, when the annealing temperature reaches 600 °C, the Ge-Sn peaks in the XRD patterns of all three samples shift to the right, suggesting a reduction in the Sn_s_ concentration within the GeSn materials. At an annealing temperature of 800 °C, the intensity of the Ge-Sn peaks becomes significantly weaker. Since VSnV defects have been experimentally confirmed as the predominant defects in as-grown GeSn layers, with other related cluster defects constituting only a minor fraction [24,25], we can reasonably conclude that at this stage, Sn_s_ has transformed into VSnV and other related clusters, with VSnV representing the dominant defect species. The XRD results demonstrate that as the annealing temperature increases, more Sn_s_ converts into VSnV, leading to a decrease in Sn_s_ concentration and an increase in VSnV concentration within the GeSn alloys.

**Figure 4:**
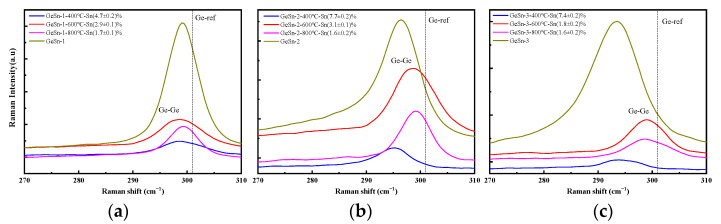
(**a**) The Raman spectrum of the GeSn/Si(001) sample 1 at different annealing temperatures; (**b**) the Raman spectrum of the GeSn/Si(001) sample 2 at different annealing temperatures; (**c**) the Raman spectrum of the GeSn/Si(001) sample 3 at different annealing temperatures.

Figure 4 presents the Raman spectra of samples 1, 2, and 3 at different annealing temperatures. It can be observed that at an annealing temperature of 400 °C, the Ge-Ge vibrational peaks of samples 1 and 2 remain unchanged, while the peak position of sample 3 shifts to the right. This further confirms that GeSn alloy samples with a higher Sn concentration exhibit inferior thermal stability compared to those with a lower Sn concentration. However, when the annealing temperature reaches 600 °C, the Ge-Ge peaks in the Raman spectra of all three samples shift to the right. At an annealing temperature of 800 °C, the intensity of the Ge-Ge peaks becomes significantly weaker. This indicates that higher annealing temperatures promote the transformation of more Sn_s_ into VSnV. These findings are consistent with the XRD analysis. Based on the shifts in the Ge-Ge vibrational peaks, the Sn_s_ concentrations of these three samples before and after annealing were calculated. Table 1 presents the XRD and Raman spectroscopy measurements along with their associated errors.

**Figure 5:**
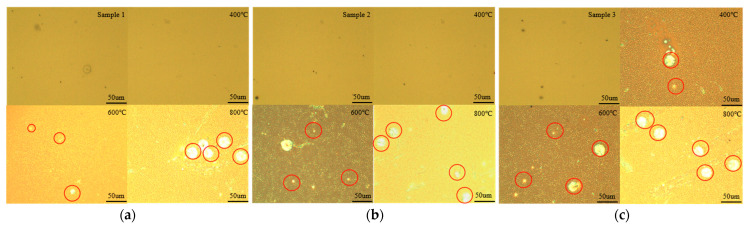
(**a**) Optical microscope images of sample 1 at different annealing temperatures; (**b**) optical microscope images of sample 2 at different annealing temperatures; (**c**) optical microscope images of sample 3 at different annealing temperatures. The red circles indicate Sn clusters.

**Figure 6:**
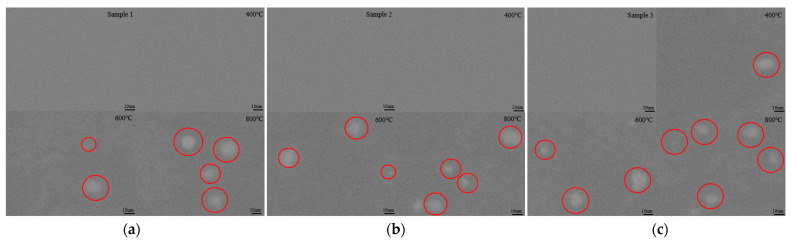
(**a**) SEM images of sample 1 at different annealing temperatures; (**b**) SEM images of sample 2 at different annealing temperatures; (**c**) SEM images of sample 3 at different annealing temperatures. The red circles indicate Sn clusters.

Figure 5 presents the optical microscope images of samples 1, 2, and 3 at different annealing temperatures, with a scale bar of 50 μm, while Figure 6 presents the scanning electron microscope (SEM) images of samples 1, 2, and 3 at different annealing temperatures, with a scale bar of 10 μm. It can be observed that samples 1 and 2 exhibit excellent stability at 400 °C, with no noticeable Sn segregation observed on the surface. When the annealing temperature reaches 600 °C, small Sn segregation points begin to appear on the surface of the samples. As the annealing temperature increases to 800 °C, these small Sn points aggregate into larger clusters. For sample 3, at 400 °C, small Sn particles are already present on the surface, indicating partial Sn segregation. When the annealing temperature reaches 600 °C, Sn clusters begin to form on the surface. At 800 °C, the number of these Sn clusters increases significantly. Therefore, the optical microscope and SEM results demonstrate that higher temperatures and higher Sn concentrations promote Sn segregation, which is consistent with the findings from the XRD and Raman analyses, as well as with results reported in other studies [28,29].

**Figure 7:**
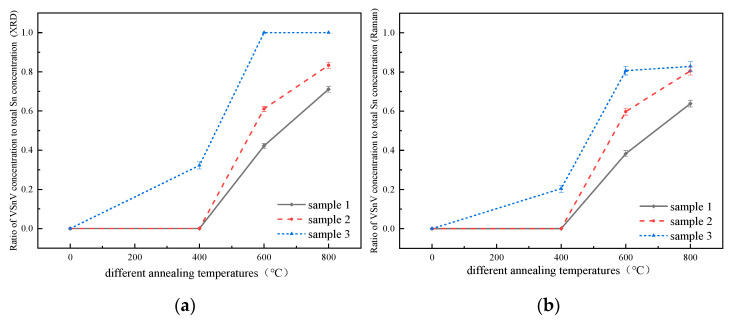
(**a**) Variation in the ratio of VSnV concentration to total Sn concentration for GeSn alloys with different Sn concentration, as determined by XRD analysis after annealing (with the error bars); (**b**) variation in the ratio of VSnV concentration to total Sn concentration for GeSn alloys with different Sn concentration, as determined by Raman analysis after annealing (with the error bars).

According to the law of mass conservation, the Sn_s_ concentration before annealing is equal to the sum of the Sn_s_ and VSnV concentration after annealing. Based on the data in Table 1, we calculated the ratio α of VSnV concentration to the initial Sn_s_ concentration for the three GeSn alloy samples after annealing, as illustrated in Figure 7. It can be observed that for the same sample, α increases with higher annealing temperatures. Additionally, at the same annealing temperature, α is larger for samples with a higher initial Sn_s_ concentration. These findings are in excellent agreement with the theoretical results reported by Ventura et al. [20]. Therefore, the analysis demonstrates that both the temperature and total Sn concentration not only influence the VSnV concentration but also affect the ratio of VSnV to the total Sn concentration.

### 2.2. The Formation Energy of Sn and VSnV

**Figure 8:**
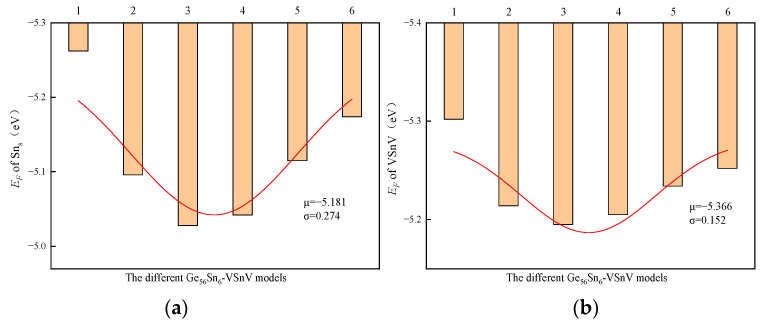
(**a**) The variation in the formation energy of Sn_s_ with different Ge_56_Sn_6_-VSnV models; (**b**) the variation in the formation energy of VSnV with different Ge_56_Sn_6_-VSnV models. The red curve represents the fitted normal distribution.

To explore the underlying physical mechanisms behind the observed experimental phenomena, we employed first-principles calculations based on DFT to investigate the formation energies, binding energies, and charge density distributions of Sn_s_ and VSnV in GeSn alloys with varying total Sn concentrations. For the selection of GeSn supercells, we employed the Special Quasi-random Structure (SQS) method to generate six distinct GeSn models with random atomic configurations for each concentration. The formation energies of both Sn_s_ and VSnV defects were calculated for each model configuration. Taking Ge_56_Sn_6_-VSnV as an example, six supercell models were generated using SQS, and the calculated formation energies of Sn_s_ and VSnV are presented in Figure 8. The results demonstrate minimal standard deviation (σ) among the different model configurations, indicating that the SQS-generated models effectively represent the random distribution of Sn atoms in the Ge matrix. The final reported values represent the average of these calculations (μ), and this methodology was consistently applied throughout all subsequent computational analyses.

**Figure 9:**
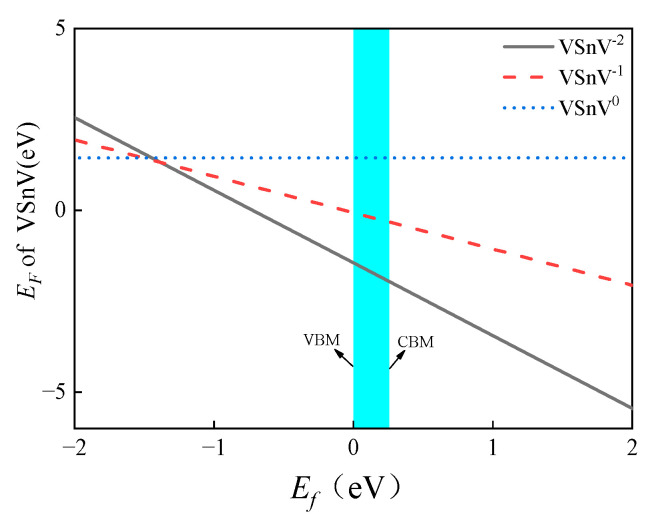
*E_F_* of a VSnV in three charge states. The blue region represents the bandgap area.

The literature indicates that a VSnV may have three charge states (0, −1, and −2) in the GeSn lattice [23], and its *E_F_* in various charge states linearly varies with the Fermi energy (*E_f_*). In the Ge_56_Sn_6_-VSnV model, we calculate the *E_F_* of the VSnV in the three charge states as a function of *E_f_*, as depicted in Figure 9. For undoped and doped GeSn, the *E_f_* is generally near the Valence Band Maximum (VBM) [30,31]. The results demonstrate that the −2 charge state of VSnV exhibits the lowest formation energy at the VBM position, which is consistent with findings from other studies [23].

For the selection of VSnV defect positions, we constructed VSnV defect models by substituting one Sn atom in the SQS-generated GeSn models. In cases where multiple Sn atoms were present in the model, each Sn atom was sequentially selected to create distinct VSnV defect configurations. The final computational results were obtained by statistically averaging all calculated VSnV configurations. For instance, in the Ge_57_Sn_5_-VSnV model, the six Sn atoms occupy different lattice positions, resulting in six possible VSnV configurations. We performed calculations for all six configurations and adopted their averaged values as the final results, as presented in Table 2. The data reveal minimal standard deviations in the formation energies of both Sn_s_ and VSnV defects, indicating that the specific location of VSnV has negligible influence on the computational outcomes.

Table 3 illustrates the variation in the formation energies of Sn_s_ and VSnV as a function of the total Sn concentration. It can be observed that as the total Sn concentration increases, the formation energy of Sn_s_ rises, while that of VSnV decreases. This indicates that the formation of Sn_s_ becomes increasingly difficult, whereas the formation of VSnV becomes more favorable with higher total Sn concentration. These computational results are consistent with our experimental findings.

**Figure 10:**
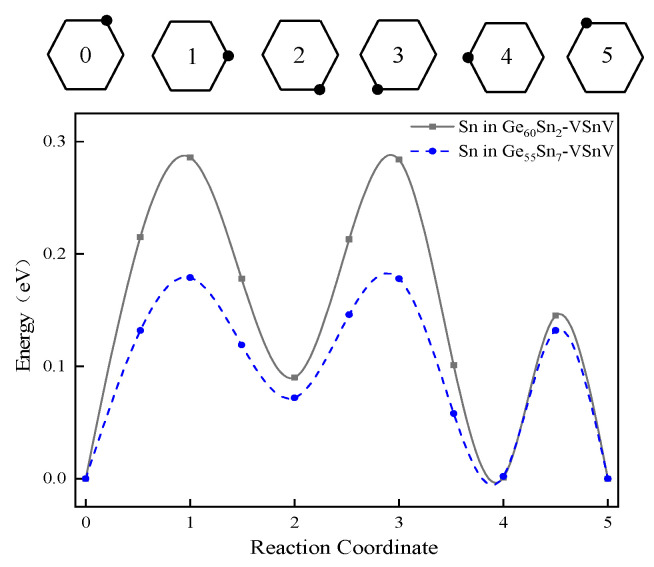
Diffusion path of the Sn using the NEB technique. At the top of the figure is the ring mechanism of diffusion for the Sn projected onto the surface of Ge. State 0 represents the initial state, state 5 represents the final state, while states 1 through 4 correspond to intermediate states.

To investigate the process of Sn surface segregation, we examined the migration kinetics of Sn atoms. Previous studies have demonstrated that Sn migrates via a vacancy-mediated mechanism [32,33,34]. Accordingly, we first constructed the initial and final configurations of Sn migration, corresponding to Configuration 0 and Configuration 5 in Figure 10. Subsequently, the nudged elastic band (NEB) method was employed to determine the intermediate transition states, followed by calculations of the migration energy barriers for Sn. Figure 10 presents the relative energy profiles for the vacancy-mediated migration of Sn atoms in Ge_60_Sn_2_-VSnV and Ge_55_Sn_7_-VSnV; the migration energy barriers were determined to be 0.286 eV and 0.179 eV for Ge_60_Sn_2_-VSnV and Ge_55_Sn_7_-VSnV, respectively. These results indicate that higher Sn concentrations lead to lower migration energy barriers, facilitating Sn atom mobility. Using the activation energy formula from references [23,35], we calculated the activation energies for Sn in Ge_60_Sn_2_-VSnV and Ge_55_Sn_7_-VSnV as 0.927 eV and 0.832 eV, respectively. This demonstrates that increased Sn concentrations result in lower activation energies, making Sn atoms more prone to displacement from their equilibrium positions. Consequently, higher Sn concentrations promote the enhanced surface segregation of Sn, which is consistent with our experimental observations.

**Figure 11:**
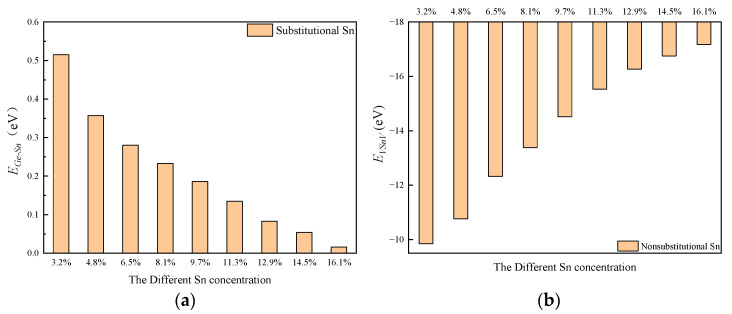
(**a**) *E*_Ge-Sn_ with the increase in total Sn concentration; (**b**) *E_VSnV_* with the increase in total Sn concentration.

To better explain the variation in the formation energies of Sn_s_, VSnV, and the migration kinetics of Sn atoms, we calculated the binding energies of Ge-Sn and VSnV, as shown in Figure 11. It can be observed that as the total Sn concentration increases, the binding energy of Ge-Sn decreases, indicating a weakening of the Ge-Sn bond and a reduction in structural stability. Consequently, a higher formation energy is required to maintain a stable configuration. Conversely, the binding energy of VSnV increases, suggesting that it requires a lower formation energy to remain stable.

**Figure 12:**
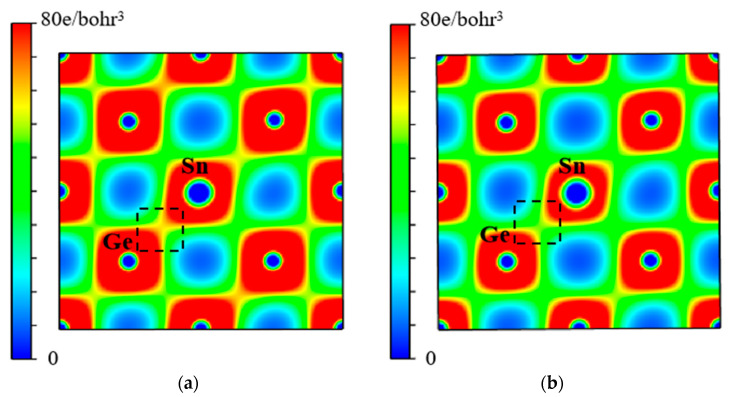
(**a**) The electron density distribution of the Ge-Sn bond at the same position in Ge_60_Sn_2_-VSnV; (**b**) the electron density distribution of the Ge-Sn bond at the same position in Ge_57_Sn_5_-VSnV.

**Figure 13:**
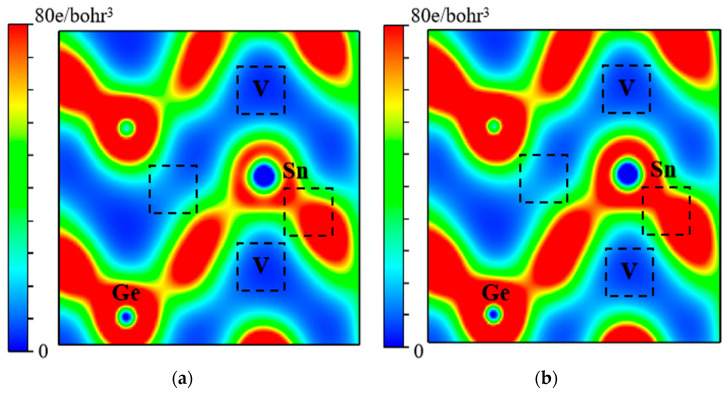
(**a**) The electron density distribution of VSnV at the same position in Ge_60_Sn_2_-VSnV; (**b**) the electron density distribution of VSnV at the same position in Ge_57_Sn_5_-VSnV.

The strength of the Ge-Sn bond and the binding of VSnV can also be intuitively reflected through the electron density distribution. Figure 12 and Figure 13 present the charge density distributions of the Ge-Sn and VSnV in Ge_60_Sn_2_-VSnV and Ge_57_Sn_5_-VSnV, respectively. It can be observed that as the total Sn concentration increases, the charge density around the Ge-Sn bond significantly decreases (indicated by the reduction in red regions), suggesting a weakening of the Ge-Sn bond. This modification demonstrates that higher Sn concentrations reduce the constraining forces exerted by the Ge matrix on Sn atoms, thereby substantially enhancing their mobility through the lattice. In contrast, the charge density around VSnV increases noticeably (indicated by the expansion of red regions and reduction in blue regions), indicating the enhanced binding capability of VSnV. These observations are in excellent agreement with the calculated results for the binding energy of Ge-Sn and VSnV discussed earlier.

## 3. Materials and Methods

### 3.1. Experimental Preparation and Characterization Methods

The GeSn thin films were grown on Si (100) substrates using an Octoplus (Dr. Eberl MBE-Komponenten GmbH, Weil der Stadt, Germany) 500 EBV MBE system, with the pressure maintained below 10^−10^ Torr. Initially, the substrate heater temperature was raised to 300 °C to initiate the epitaxial growth of a Ge buffer layer, which was deposited to a thickness of 30 nm. Subsequently, the substrate temperature was reduced to 180 °C to commence the growth of the GeSn thin films. The GeSn layers were formed through the co-deposition of Ge and Sn beams, achieving a thickness of 100 nm. The concentration of the alloy was controlled by adjusting the beam intensities of Ge and Sn. To investigate the thermal stability and Sn surface segregation after annealing, the samples were sectioned and subjected to rapid thermal annealing (RTA) at temperatures of 400 °C, 600 °C, and 800 °C for a duration of 60 s.

In this study, we employed a multi-technique analytical approach to comprehensively characterize our experimental results: XRD measurements served as the primary quantitative method, and Raman spectroscopy provided semi-quantitative data, while optical microscope and SEM observations offered qualitative assessments. This complementary combination of four characterization techniques enabled the robust analysis of the experimental findings. Furthermore, to minimize measurement uncertainties, we conducted multiple measurements on the samples using XRD and Raman spectroscopy to ensure data reliability.

The concentration of Sn_s_ in GeSn was analyzed using XRD. Measurements were performed in the (004) and (224) 2θ-ω scan directions to determine the lattice constants. The formulas used to calculate the Sn_s_ concentration from the XRD data are given by Equations (1) and (2) [36,37].

(1) $$a_{0}=\frac{a_{⊥}+\frac{2C_{12}}{C_{11}}a_{//}}{1+\frac{2C_{12}}{C_{11}}}=\frac{\lambda }{1+\frac{2C_{12}}{C_{11}}}\left(\frac{2}{\sin\theta_{004}}+\frac{2C_{12}}{C_{11}}\frac{\sqrt{2}}{\sqrt{\sin^{2}\theta_{224}-\sin^{2}\theta_{004}}}\right)$$

(2) $$a_{0}=a_{\mathrm{Ge}}(1-x)+a_{\mathrm{Sn}}x+bx(1-x)$$

Here, *C*_12_ and *C*_11_ represent the elastic constants, and the lattice constants perpendicular to the plane (*a*_⊥_) and in-plane (*a_∥_*) are obtained from the (004) and (224) XRD curves. *a*_Ge_ and *a*_Sn_ represent the lattice constants of Ge and Sn, respectively, and *b* is the bending coefficient.

The concentration of Sn_s_ in GeSn alloys can also be calculated using Raman spectroscopy, as described by Equation (3) [15,38,39,40,41,42]. Optical microscope and SEM were employed to observe the surface segregation of Sn in GeSn before and after annealing.

(3) $$\Delta \omega =\Delta \omega_{\mathrm{composition}}x+\Delta \omega_{\mathrm{strain}}\varepsilon_{//}$$

where $\Delta \omega_{\mathrm{composition}}$ is the concentration-dependent frequency shift coefficient of the Ge-Ge peak, and $\Delta \omega_{\mathrm{strain}}$ is the strain-dependent frequency shift coefficient of the Ge-Ge peak. $\varepsilon_{//}$ represents the in-plane strain of the material. Based on the literature [39], the corresponding strain correlation coefficients were obtained, and the strain values were subsequently calculated.

### 3.2. First-Principles Calculation Methods and Model

**Figure 14:**
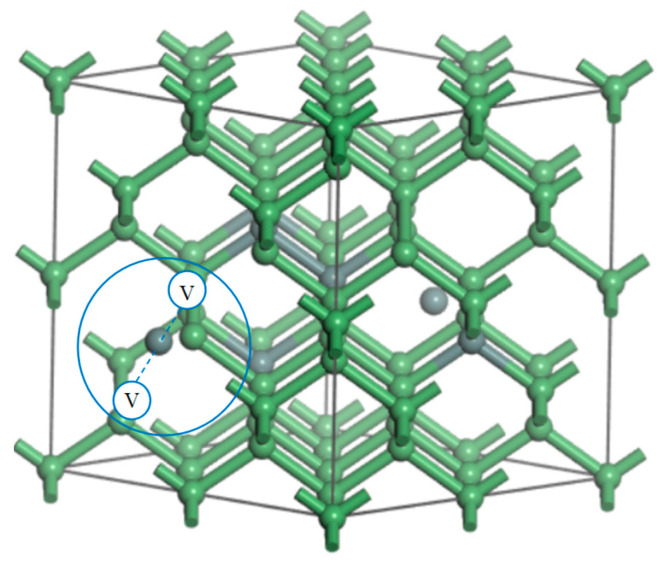
Configuration diagram of Ge_57_Sn_5_-VSnV (the green, gray, and white balls are Ge, Sn, and V, respectively).

In the theoretical study, a supercell model of Ge_1−*x*_Sn*_x_*-VSnV was employed. The SQS method was utilized to achieve a random distribution within appropriately sized supercells, and the total Sn concentration in Ge_1−*x*_Sn*_x_*-VSnV was simulated by varying the number of Sn atoms [43]. Taking a 64-atom supercell as an example, the total Sn concentration was increased from Ge_60_Sn_2_-VSnV to Ge_52_Sn_10_-VSnV in models containing VSnV. For instance, the Ge_57_Sn_5_-VSnV model consists of 57 Ge atoms, 5 Sn_s_ atoms, and one VSnV defect, as illustrated in Figure 14. In these models, green spheres represent Ge atoms, while gray spheres represent Sn atoms. To enhance computational reliability, we employed the SQS method to generate six distinct GeSn models with randomized atomic configurations for each alloy concentration. Subsequently, we calculated the formation energies of both Sn_s_ and VSnV defects for each individual model configuration. The final reported values represent the statistical averages of these multiple calculations, thereby improving the robustness of our computational results. In this section, we employed first-principles computational methods. The plane-wave cut-off energy was set to 300 eV, and a 4 × 4 × 4 k-point mesh density was adopted for Brillouin zone sampling according to the Monkhorst–Pack scheme. Electron exchange and correlation were described using the Perdew–Burke–Ernzerhof (PBE) functional. For Ge and Sn, the projector augmented wave (PAW) method was used to approximate the core electron states, the [Ar] 3d^10^ and [Kr] 4d^10^ state, whereas the 4s^2^ 4p^2^ and 5s^2^ 5p^2^ states were treated as valence electrons. The convergence criteria for forces and total energy in self-consistent calculations were set to 0.001 eV/Å and 1 × 10^−6^ eV, respectively. All calculations were performed using the VASP 5.4 software package. SQSs were generated using the SQS module in the Alloy Theoretic Automated Toolkit (ATAT) software package (https://www.brown.edu/Departments/Engineering/Labs/avdw/atat/, accessed on 14 April 2025), the energy barriers were obtained using the NEB method for finding the transition state, and the electron density distribution maps were visualized using VESTA.

The formation energies of Sn_s_ and VSnV in GeSn alloys with varying Sn concentrations are expressed by Equation (4) [44].

(4) $$E_{F}=E(a,q)-E(host)+\sum_{i}n_{i}(E_{i}+\mu_{i})+q\left[E_{\mathrm{VBM}}(host)+E_{f}+△V\right]$$

where *E*(*a*, *q*) represents the total energy of the supercell containing defect *a* with charge *q*, *E*(*host*) is the total energy of the defect-free supercell, *n_i_* denotes the number of atoms added or removed to create defect a, *E_i_* is the elemental material energy, *μ_i_* is its chemical potential in the environment relative to elemental material, *E*_VBM_(*host*) is the valence band maximum energy of the perfect supercell, *E_f_* is the Fermi level of the material, and Δ*V* is the difference in the average electrostatic potential between the defective supercell and the perfect supercell.

The binding energy of the Ge-Sn bond in GeSn alloys with a varying Sn concentration is expressed by Equation (5) [45].

(5) $$E_{\mathrm{Ge}\text{-}\mathrm{Sn}}=(nE_{\mathrm{Ge}}+mE_{\mathrm{Sn}}-E_{\mathrm{total}}-pE_{\mathrm{Ge}\text{-}\mathrm{Ge}}-\;tE_{\mathrm{Sn}\text{-}\mathrm{Sn}})/r$$

where *E*_total_ represents the total energy of the supercell system, *E*_Ge_ denotes the energy of a single Ge atom, *E*_Sn_ denotes the energy of a single Sn atom, *E*_Ge-Ge_ represents the binding energy of the Ge-Ge bond, *E*_Sn-Sn_ represents the binding energy of the Sn-Sn bond, *n* is the number of Ge atoms, *m* is the number of Sn atoms, *p* is the number of Ge-Ge bonds, *t* is the number of Sn-Sn bonds, and *r* is the number of Ge-Sn bonds.

The binding energy of VSnV in GeSn alloys with varying Sn concentrations is expressed by Equation (6) [46].

(6) $$E_{\mathrm{VSnV}}=2E_{F}(V)+E_{F}(\mathrm{Sn})-E_{F}(\mathrm{VSnV})$$

where *E_F_*(V) is the formation energy of the vacancy, *E_F_*(Sn) is the formation energy of Sn_s_, and *E_F_*(VSnV) is the formation energy of the VSnV.

## 4. Conclusions

To investigate the relationship between VSnV concentration and total Sn concentration, we first prepared GeSn materials with varying Sn concentrations using MBE and subjected them to annealing at different temperatures. Subsequently, the VSnV concentration and Sn surface segregation were characterized. We employed XRD for quantitative analysis, incorporating repeated measurements to account for experimental errors. The XRD results demonstrate that both higher Sn concentrations and elevated temperatures lead to increased VSnV concentrations, with VSnV constituting a progressively larger proportion of the total Sn concentrations. These findings were corroborated by semi-quantitative Raman spectroscopic analysis. Subsequent qualitative characterization through an optical microscope and SEM demonstrated pronounced Sn surface segregation under conditions of increased temperature and Sn concentrations, exhibiting excellent agreement with both the XRD/Raman experimental data and established theoretical results. To explain these phenomena, we employed first-principles calculations based on DFT to determine the formation energies, binding energies, and charge density distributions of Sn_s_ and VSnV in GeSn alloys. The results indicate that as the total Sn concentration increases, the formation energy of Sn_s_ rises, while that of VSnV decreases, and both the migration energy barrier and activation energy of Sn are reduced, suggesting that VSnV becomes more favorable to form and Sn is easy to migrate. The binding energy and electron density distribution of Ge-Sn reveal that increasing total Sn concentration leads to the progressive weakening of Ge-Sn bonds, necessitating greater energy input to maintain structural stability and significantly enhancing Sn atomic mobility. Meanwhile, the binding energy and electron density distribution of VSnV demonstrate that its binding capability strengthens with increasing total Sn concentration, thereby reducing the energy required for its formation. This study provides both theoretical and experimental references for GeSn material growth and VSnV defect control through a combined theoretical–experimental approach, offering significant guidance for enhancing device performance. The relevant theoretical and experimental methodologies can be extended to similar studies in other group IV alloys.

## Figures and Tables

**Table 1:** The Sn_s_ concentration measured by XRD and Raman spectroscopy.

Annealing Temperature	The Sn_s_ Concentration Measured by XRD			The Sn_s_ Concentration Measured by Raman Spectroscopy		
	Sample 1	Sample 2	Sample 3	Sample 1	Sample 2	Sample 3
None	(4.50 ± 0.07)%	(7.20 ± 0.09)%	(8.6 ± 0.1)%	(4.7 ± 0.2)%	(7.7 ± 0.2)%	(9.3 ± 0.3)%
400	(4.50 ± 0.09)%	(7.20 ± 0.16)%	(6.6 ± 0.1)%	(4.7 ± 0.2)%	(7.7 ± 0.2)%	(7.4 ± 0.2)%
600	(2.6 ± 0.1)%	(2.80 ± 0.09)%	0	(2.9 ± 0.1)%	(3.1 ± 0.1)%	(1.8 ± 0.2)%
800	(1.30 ± 0.06)%	(1.20 ± 0.06)%	0	(1.7 ± 0.1)%	(1.6 ± 0.2)%	(1.6 ± 0.2)%

**Table 2:** The variation in the formation energy of Sn_s_ and VSnV with different VSnV locations in Ge_57_Sn_5_-VSnV models.

The Ge_57_Sn_5_-VSnV Models with Different VSnV Locations	1	2	3	4	5	6	μ	σ
*E_F_* of Sn_s_ (eV)	−5.252	−5.169	−5.183	−5.217	−5.237	−5.216	−5.212	0.028
*E_F_* of VSn (VeV)	−5.117	−5.028	−5.209	−5.097	−5.059	−5.257	−5.128	0.279

**Table 3:** The variation in the formation energy of Sn_s_ and VSnV with the increase in total Sn concentration.

The Total Sn Concentration	3.2%	4.8%	6.5%	8.1%	9.7%	11.3%	12.9%	14.5%	16.1%
*E_F_* of Sn_s_ (eV)	−5.814	−5.691	−5.318	−5.212	−5.188	−5.146	−5.018	−4.976	−4.873
*E_F_* of VSnV (eV)	−4.244	−4.403	−4.837	−5.128	−5.374	−5.528	−5.783	−5.916	−6.279

## Data Availability

Data are contained within the article.
